# Optimizing treatment for Gleason 10 prostate cancer: radiation dose escalation and ^68^Ga-PSMA-PET/CT staging

**DOI:** 10.1007/s00066-025-02376-1

**Published:** 2025-02-28

**Authors:** Cem Onal, Ozan Cem Guler, Birhan Demirhan, Petek Erpolat, Aysenur Elmali, Melek Yavuz

**Affiliations:** 1 Department of Radiation Oncology, Faculty of Medicine, Adana Dr. Turgut Noyan Research and Treatment Center, Baskent University, 01120 Adana, Turkey; 2 Department of Radiation Oncology, Faculty of Medicine, Baskent University, Ankara, Turkey; 3 Division of Radiation Oncology, Iskenderun Gelisim Hospital, Hatay, Turkey; 4 Department of Radiation Oncology, Faculty of Medicine, Gazi University, Ankara, Turkey

**Keywords:** Prostate cancer, Radiotherapy, Androgen deprivation therapy, Gleason score, Survival

## Abstract

**Purpose:**

This study aimed to investigate the effects of dose escalation through focal boost (FB) to intraprostatic lesions (IPLs) as well as the role of gallium-68 prostate-specific membrane antigen positron-emission tomography (^68^Ga-PSMA-PET/CT) for staging and treatment planning in patients with Gleason score (GS) 10 prostate cancer (PCa) receiving definitive radiotherapy (RT) and androgen deprivation therapy (ADT).

**Materials and methods:**

We retrospectively analyzed data of 92 patients with GS 10 PCa who underwent definitive RT and ADT from March 2010 to October 2022. Freedom from biochemical failure (FFBF), prostate cancer-specific survival (PCSS), distant metastasis-free survival (DMFS), and overall survival (OS) rates were calculated using the Kaplan–Meier method. Survival outcomes were compared between patients staged with ^68^Ga-PSMA-PET/CT and those staged with conventional imaging modalities as well as between those who received a simultaneous integrated boost (SIB) and those who did not.

**Results:**

At a median follow-up time of 73 months, the 5‑year FFBF, PCSS, DMFS, and OS rates were 59.2%, 77.0%, 62.9%, and 67.6%, respectively. Disease progression was observed in 39 patients (42.4%), with most cases manifesting as distant metastasis (DM). A total of 56 patients (60.9%) were staged using ^68^Ga-PSMA-PET/CT, while 43 patients (46.7%) received FB to IPLs. Patients staged with ^68^Ga-PSMA-PET/CT had better FFBF and PCSS compared to those staged with conventional imaging. Patients undergoing an SIB had improved PCSS and DMFS. In the multivariable analysis, an ADT duration of 18 months or more was associated with improved FFBF, PCSS, DMFS, and OS. Application of an SIB was an additional independent predictor for improved FFBF, while staging with ^68^Ga-PSMA-PET/CT was associated with better PCSS.

**Conclusion:**

We found that long-term ADT, increasing the radiation dose to primary tumor, and staging with ^68^Ga-PSMA-PET/CT improved clinical outcomes. Additional research is needed for validation.

## Introduction

Prostate cancer (PCa) classified as Gleason grade 5 includes cases with Gleason scores (GS) of 9 and 10, exhibiting an incidence of 9–16% [1, 2]. GS PCa 10 is an uncommon and poorly differentiated form of cancer, known for its aggressive characteristics and historically unfavorable prognosis [3, 4].

While the precise management of and treatment outcomes for GS 10 PCa remain uncertain due to its infrequency, a therapeutic approach involving a combination of radiotherapy (RT) and androgen deprivation therapy (ADT) has been applied in the treatment of patients diagnosed with high-risk PCa [5, 6]. In recent years, significant progress has been made in the field of RT and systemic therapy, along with the development of new functional imaging techniques. Recent studies have shown that increasing the radiation dose to areas with visible disease can yield significant benefits, especially for patients with high-risk PCa [7–9]. In addition, the clinical benefit of pelvic RT has been recently demonstrated in a randomized trial [10]. Multiple studies have shown the survival advantage of long-term ADT compared to short-term or no ADT [5, 11]. There has been a clear trend toward the use of novel molecular imaging techniques for staging purposes, specifically gallium-68 prostate-specific membrane antigen positron-emission tomography/computed tomography (^68^Ga-PSMA-PET/CT). This approach has the potential to enhance the accuracy of staging by identifying smaller nodal metastases while effectively ruling out distant metastasis (DM) [12].

Given the scarcity of pure GS 10 tumors, many studies have grouped the outcomes of patients with GS 8 and 9 tumors [13–16], and few studies have specifically assessed the treatment outcomes of patients with GS 10 PCa [17–19]. Moreover, these studies have been limited by small sample sizes, short follow-up periods, and variations in treatment approaches. In this study, we sought to investigate the effects of dose escalation using focal boost (FB) to intraprostatic lesions (IPLs) and ^68^Ga-PSMA-PET/CT for staging and RT planning in patients with GS 10 PCa undergoing definitive RT and ADT using modern RT techniques. We conducted a comparative analysis of survival outcomes between patients staged with ^68^Ga-PSMA-PET/CT and those staged using conventional imaging modalities. Furthermore, we assessed survival outcomes in relation to the administration of a simultaneous integrated boost (SIB) versus non-receipt of this treatment.

## Materials and methods

### Patient selection

An analysis was conducted on the clinical data of 92 patients diagnosed with GS 10 PCa who underwent definitive RT and ADT from March 2010 to October 2022. These cases accounted for 0.65% of the PCa patients who underwent definitive RT at our institution during this period. The study included patients who had a confirmed histological diagnosis of GS 10 PCa, who had a minimum follow-up period of 24 months, and who had received ADT and external RT. Patients who had undergone radical prostatectomy (RP), who had distant metastasis, and who had received ADT for less than 6 months were excluded.

This study was approved by the Baskent University Institutional Review Board (project no. KA23/20) and supported by the Baskent University Research Fund. Written informed consent was obtained from all patients.

### Treatment planning

All patients received volumetric modulated arc therapy or intensity-modulated RT as their definitive form of treatment. The simultaneous integrated boost (SIB) technique was used to administer 78 Gy to the prostate and seminal vesicles and 86 Gy for FB to IPLs in all patients [9, 20]. Additional pelvic nodal irradiation was administered to all patients, totaling 46–54 Gy radiation dose. The daily fraction doses were 2 Gy for the prostate and pelvic nodal areas, while a fraction dose of 2.2 Gy per day was administered for the FB to intraprostatic lesions (IPLs). Focal boost to gross lymph node metastases or PSMA-positive lymph nodes was not applied.

All IPLs were defined by an experienced genitourinary radiologist using data from digital rectal examinations, pathological biopsies that included at least 12 cores from four quadrants, and the identification of focal low-signal intensity areas in the transition zones and periphery. Imaging sequences, including diffusion-weighted magnetic resonance imaging (DW-MRI), dynamic contrast-enhanced (DCE) imaging, and T2-weighted imaging, were employed to delineate the gross tumor volumes of all visible IPLs [9, 20].

The PSMA-PET/CT procedure utilizes a radioactive tracer known as gallium-68 PSMA. All patients underwent the ^68^Ga-PSMA-PET/CT scan using the same tracer. ^68^Ga-PSMA-PET/CT is used for evaluating nodal and distant metastases; however, it is not routinely employed to delineate IPLs.

ADT consisting of luteinizing hormone-releasing hormone agonists alone (LHRHA) or in combination with an anti-androgen was administered to all patients.

### Follow-up

Patients were monitored every 3 months for 2 years. After that, monitoring occurred every 6 months until the fifth year and then annually thereafter. Biochemical failure (BF) was defined based on the Phoenix criteria (PSA nadir plus 2 ng/mL) [21]. Imaging evaluations that identified the occurrence of locoregional recurrence (LR) or DM were used to determine clinical progression. LR refers to any recurrence that occurs in the prostate and/or regional lymph nodes. Meanwhile, DM pertains to recurrences in non-regional lymph nodes, excluding those in the pelvic region.

Toxicities were categorized as acute, defined as those occurring within 90 days of initiating radiotherapy (RT), or late, defined as those observed more than 90 days after RT initiation. Gastrointestinal (GI) and genitourinary (GU) toxicities were assessed using the Common Terminology Criteria for Adverse Events version 4.0.2.

### Statistical analysis

Statistical analysis was performed using SPSS version 26.0 (SPSS for Windows; IBM Corp., Armonk, NY, USA) and GraphPad Prism version 10.1.2 (GraphPad Software; Boston, MA, USA). The endpoints examined included FFBF, which represents the duration from diagnosis to BF; PCSS, which represents the duration from diagnosis to cancer-related death or most recent follow-up; DMFS, which represents the duration from diagnosis to DM; and OS, which is calculated by subtracting the diagnosis date from the date of death or last follow-up. The FFBF, PCSS, DMFS, and OS rates were calculated using the Kaplan–Meier method. The log-rank test was used for the univariate analysis. Covariates with *p*-values < 0.05 in univariate analysis were included in the Cox proportional hazards model for the multivariate analyses. All *p*-values less than 0.05 was considered statistically significant.

## Results

Table 1 provides an overview of the patient and tumor characteristics for the entire patient population. The cohort had a median age of 66 years (range 45–85 years). A significant proportion of patients (72.8%) presented with clinical T3a stage disease or higher, while more than half (53.3%) exhibited regional lymph node metastasis. Patients underwent ADT for a median duration of 24 months (range 8–38 months). The median duration of ADT for patients receiving treatment for less than 18 months was 15 months (range 8–16 months), whereas it was 24 months (range 18–38 months) for those undergoing ADT for 18 months or more. Almost half of the patients (46.7%) underwent a FB to IPLs using the SIB technique. The median total prostate and pelvic field doses were 78 Gy (range 76–86 Gy) and 54 Gy (range 46–54 Gy), respectively.

**Table 1:** Patient and tumor characteristics

Characteristics	Number	%
*Age, years, median (range)*	66 (45–85)	
*PSA, ng/mL, median (range)*	26.2 (2.7–881.7)	
*T stage*		
T2b	10	10.9
T2c	15	16.3
T3a	24	26.1
T3b	43	46.7
*N stage*		
N0	49	53.3
N1	43	46.7
*PSMA-PET/CT*		
Present	56	60.9
Absent	36	39.1
*SIB*		
Present	43	46.7
Absent	49	53.3
*ADT duration*		
< 18 months	21	22.8
≥ 18 months	71	77.2

*ADT* androgen deprivation therapy, *PSA* prostate-specific antigen, *PSMA-PET/CT* prostate-specific membrane antigen positron-emission tomography, *SIB* simultaneous integrated boost

**Fig. 1:**
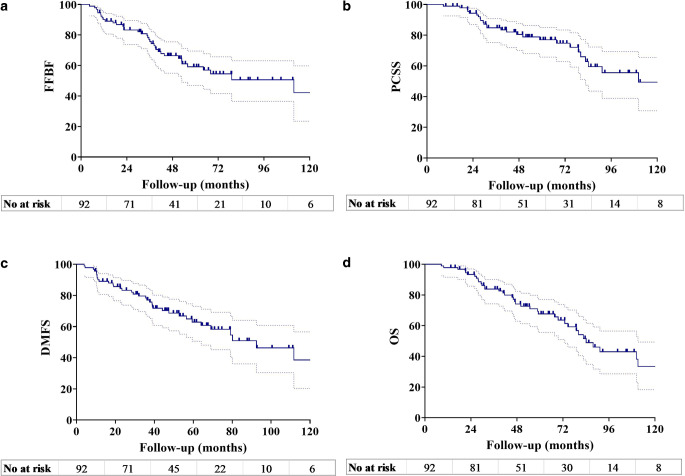
Kaplan–Meier plots of **a** freedom from biochemical failure (*FFBF*), **b** prostate cancer-specific survival (*PCSS*), **c** distant metastasis-free survival (*DMFS*), and **d** overall survival (*OS*) of the entire cohort

The median follow-up time in this study was 73.0 months (59.1–86.7 months; 95% interquartile range [IQR]). The 5‑year FFBF, PCSS, DMFS and OS rates were 59.2%, 77.0%, 62.9%, and 67.6%, respectively (Fig. 1). Disease progression was observed in 39 patients (42.4%): 31 patients (33.7%) with DM only, 3 patients (3.3%) with LR only, and 5 patients (5.5%) with both DM and LR. At the time of last follow-up, 53 patients (57.6%) were alive (13 patients [14.1%] had disease) and 39 patients (42.4%) had died (27 patients [29.3%] had disease and 12 patients [13.1%] did not).

The prognostic factors for FFBF in univariable analysis included ^68^Ga-PSMA-PET/CT staging, SIB use, and duration of ADT (Table 2). For PCSS, clinical T and N stages, ^68^Ga-PSMA-PET/CT staging, and ADT duration were significant prognostic factors. In univariable analysis, clinical T stage and ADT duration were significant prognostic factors for DMFS and OS (Table 3). An SIB was identified as an additional prognostic factor for DMFS.

**Table 2:** Univariable and multivariable analyses of the prognostic factors for freedom from biochemical failure (FFBF) and prostate cancer-specific survival (PCSS) of the entire cohort

	FFBF				PCSS			
	Univariate analysis		Multivariate analysis		Univariate analysis		Multivariate analysis	
Characteristic	HR (95% CI)	*p*-value	HR (95% CI)	*p-*value	HR (95% CI)	*p*-value	HR (95% CI)	*p*-value
Age	1.01 (0.97–1.05)	0.57	–	–	1.04 (0.99–1.09)	0.16	–	–
PSA	0.70 (0.36–1.38)	0.3	–	–	0.74 (0.22–1.66)	0.46	–	–
T stage, < T3a vs.≥ T3a	0.44 (0.18–1.08)	0.07	–	–	0.10 (0.01–0.72)	0.02	0.19 (0.02–1.49)	0.11
N stage, N0 vs. N1	0.62 (0.33–1.19)	0.15	–	–	0.44 (0.20–0.95)	0.04	0.79 (0.33–1.90)	0.6
PSMA-PET/CT, + vs. −	0.44 (0.23–0.88)	0.02	0.67 (0.28–1.60)	0.37	0.35 (0.14–0.85)	0.02	0.38 (0.15–0.95)	0.04
SIB, + vs. −	0.40 (0.19–0.85)	0.02	0.46 (0.21–0.91)	0.04	0.39 (0.14–1.04)	0.06	–	–
ADT duration, < 18 months vs.≥ 18 months	0.28 (0.14–0.56)	< 0.001	0.26 (0.21–0.91)	< 0.001	0.15 (0.07–0.34)	< 0.001	0.15 (0.06–0.36)	< 0.001

*ADT* androgen deprivation therapy, *CI* confidence interval, *FFBF* freedom from biochemical failure, *HR* hazard ratio, *PCSS* prostate cancer-specific survival, *PSA* prostate-specific antigen, *PSMA-PET/CT* prostate-specific membrane antigen positron-emission tomography, *SIB* simultaneous integrated boost

**Table 3:** Univariable and multivariable analyses of the prognostic factors for distant metastasis-free survival (DMFS) and overall survival (OS) of the entire cohort

	DMFS				OS			
	Univariate analysis		Multivariate analysis		Univariate analysis		Multivariate analysis	
Characteristic	HR (95% CI)	*p*-value	HR (95% CI)	*p*-value	HR (95% CI)	*p*-value	HR (95% CI)	*p*-value
Age	1.00 (0.96–1.05)	0.84	–	–	1.03 (0.99–1.08)	0.16	–	–
PSA	0.67 (0.33–1.34)	0.26	–	–	1.13 (0.59–2.14)	0.72	–	–
T stage, < T3a vs.≥ T3a	0.37 (0.14–0.95)	0.04	0.46 (0.18–1.21)	0.12	0.37 (0.15–0.95)	0.04	0.44 (0.17–1.13)	0.09
N stage, N0 vs. N1	0.59 (0.31–1.15)	0.12	–	–	0.80 (0.42–1.54)	0.51	–	–
PSMA-PET/CT, + vs. −	0.62 (0.31–1.23)	0.17	–	–	0.55 (0.27–1.12)	0.10	–	–
SIB, + vs. −	0.43 (0.20–0.92)	0.03	0.56 (0.26–1.23)	0.15	0.54 (0.25–1.15)	0.11	–	–
ADT duration, < 18 months vs.≥ 18 months	0.34 (0.17–0.68)	0.003	0.39 (0.19–0.79)	0.009	0.21 (0.11–0.41)	< 0.001	0.23 (0.12–0.45)	< 0.001

*ADT* androgen deprivation therapy, *CI* confidence interval, *DMFS* distant metastasis-free survival, *HR* hazard ratio, *OS* overall survival, *PSA* prostate-specific antigen, *PSMA-PET/CT* prostate-specific membrane antigen positron-emission tomography, *SIB* simultaneous integrated boost

**Fig. 2:**
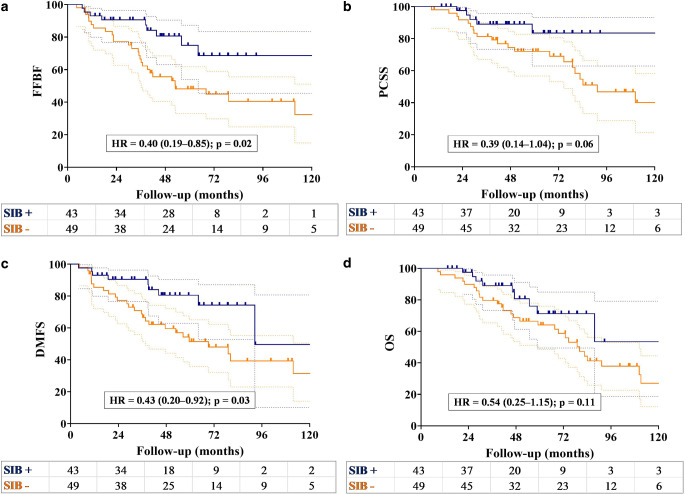
Kaplan–Meier curve of patients undergoing a simultaneous integrated boost (SIB; blue line) and those without SIB (yellow line) demonstrating **a** freedom from biochemical failure (*FFBF*), **b** prostate cancer-specific survival (*PCSS*), **c** distant metastasis-free survival (*DMFS*), and **d** overall survival (*OS*)

**Fig. 3:**
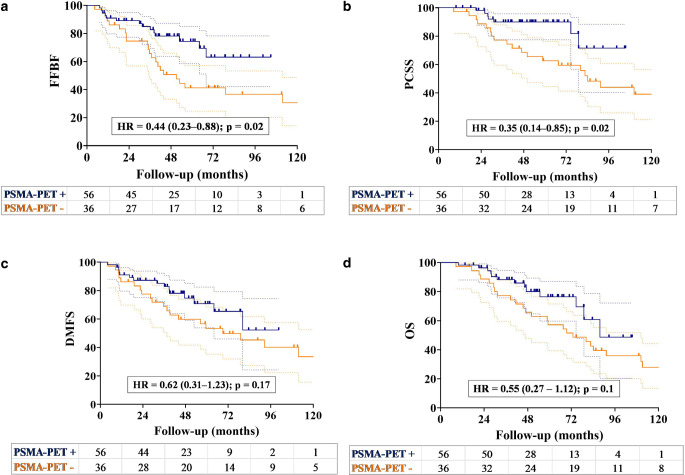
Kaplan–Meier plots of the patients who underwent 68Ga-PSMA-PET/CT (*blue line*) and conventional imaging (yellow line), demonstrating **a** freedom from biochemical failure (*FFBF*), **b** prostate cancer-specific survival (*PCSS*), **c** distant metastasis-free survival (*DMFS*), and **d** overall survival (*OS*)

Use of the SIB technique resulted in significant improvements in 5‑year FFBF (74.8% vs. 48.1%; *p* = 0.02) and DMFS rates (80.5% vs. 51.5%; *p* = 0.02; Fig. 2a, c). Additionally, there were borderline significant improvements in 5‑year PCSS (83.5% vs. 72.0%; *p* = 0.06) and OS (71.3% vs. 64.0%; *p* = 0.1) in patients receiving the SIB technique (Fig. 2b, d). There was a significant difference in the 5‑year FFBF (74.4% vs. 41.2%; *p* = 0.02) and PCSS rates (90.0% vs. 62.6%; *p* = 0.02) between patients who underwent ^68^Ga-PSMA-PET/CT and those who underwent conventional imaging modalities (Fig. 3a, b). The 5‑year DMFS rates (70.8% vs. 53.3%; *p* = 0.17) and OS rates (76.4% vs. 57.2%; *p* = 0.1) were higher in patients with ^68^Ga-PSMA-PET/CT staging compared to those without (Fig. 3c, d). However, there was no significant difference observed between the two groups.

The multivariable analysis revealed that an ADT duration of 18 months or longer is a significant predictor of improved FFBF, PCSS, DMFS, and OS. Another factor for improved FFBF was the use of the SIB technique, along with the staging of patients using ^68^Ga-PSMA-PET/CT, which led to better PCSS. The multivariable analysis revealed that advanced T stage had borderline significance for predicting worse PCSS, DMFS, and OS. Additionally, treatment with an SIB was associated with improved DMFS, approaching the level of significance (Table 3).

With the exception of one patient (1.1%) who experienced gross hematuria requiring cystoscopic evaluation, no other patients encountered severe adverse events that necessitated discontinuation of RT. Acute grade ≥ 2 GI toxicities were reported in 10 patients (10.9%), primarily manifesting as proctitis and diarrhea (Table 4). Additionally, 25 patients (27.2%) experienced acute grade ≥ 2 GU toxicities, mainly cystitis and urgency.

**Table 4:** Acute and late gastrointestinal (GI) and genitourinary (GU) system toxicities of the entire cohort

Toxicity type	Acute, *n* (%)	Late, *n* (%)
*Gastrointestinal*		
0	61 (66.3)	87 (94.5)
I	21 (22.8)	1 (1.1)
II	10 (10.9)	3 (3.3)
III	0	1 (1.1)
*Genitourinary*		
0	29 (31.5)	83 (90.1)
I	38 (41.3)	3 (3.3)
II	24 (26.1)	3 (3.3)
III	1 (1.1)	3 (3.3)

Late grade ≥ 2 GI toxicity was observed in 4 patients (4.4%), with 3 patients (3.3%) having grade 2 and 1 patient (1.1%) having grade 3 toxicity. Late grade ≥ 2 GU toxicity was noted in 6 patients (6.6%), with 3 patients (3.3%) experiencing grade 2 and 3 patients (3.3%) experiencing grade 3.

There were no significant differences in acute grade ≥ 2 GI toxicities (3.3% vs. 7.6%; *p* = 0.37), acute grade ≥ 2 GU toxicities (10.9% vs. 16.3%; *p* = 0.49), late grade ≥ 2 GI toxicities (0% vs. 4.3%; *p* = 0.12), or late grade ≥ 2 GU toxicities (2.2% vs. 4.3%; *p* = 0.40) between patients receiving an FB to IPLs and those who did not receive an FB.

## Discussion

The findings of this study show that GS 10 PCa is highly aggressive, as evidenced by a recurrence rate of more than 40% among patients, particularly in the form of DM, emphasizing the importance of implementing effective treatment strategies and appropriate staging. The use of long-term ADT had a significant positive impact on all treatment outcome parameters, while the use of ^68^Ga-PSMA-PET/CT for staging resulted in a significant improvement in FFBF and PCSS. Moreover, implementation of the SIB technique resulted in a significant improvement in FFBF and DMFS, while not leading to a statistically significant increase in acute or late GI and GU toxicities.

The optimal management strategies and treatment outcomes for GS 10 PCa are not well established due to its rarity. Recent database trials involving a substantial number of GS 9 and 10 cancers have shown that both RT and RP yield similar treatment results [14–16]. However, the proportion of GS 10 PCa patients in these studies was less than 10% of the study cohort. Previous studies demonstrated differences in mortality between patients with biopsied GS 9 and 10 PCa treated with RP or external beam radiation therapy (EBRT), with the highest mortality observed in GS 10 PCa patients [2, 22]. Only a few studies have evaluated the outcomes of patients with GS 10 PCa treated with different modalities [17–19]. Mai et al. [18] found that the 5‑year DMFS, PCSS, and OS rates were 57%, 67%, and 57%, respectively, for 9 patients who underwent EBRT and long-term ADT. Inman et al. [17] conducted an analysis of 13 patients who had pathologically confirmed GS 10 disease after RP. They found that the 5‑year PCSS and OS rates were 77% and 66%, respectively. Sandler et al. [19] found that aggressive therapy with curative intent is warranted, as more than half of 112 GS 10 PCa patients treated with RP (26 patients), EBRT (48 patients), and EBRT with brachytherapy (BT) boost (38 patients) remained free of systemic disease 5 years after treatment. The current study confirms previous findings, despite nearly half of the patients having nodal metastases, a risk factor for poor survival. Several factors contribute to this result, such as the use of ^68^Ga-PSMA-PET/CT for staging in over 50% of patients, the implementation of pelvic lymph node RT, the application of a higher radiation dose, and the use of ADT in all patients.

Recent developments in imaging techniques have greatly enhanced the ability to detect DM, leading to improved clinical staging [12]. Moreover, ^68^Ga-PSMA-PET/CT is frequently used for RT planning in high-risk PCa patients. Multiple studies have demonstrated that the outcomes of ^68^Ga-PSMA-PET/CT often have a significant impact on the course of local RT [23, 24]. Approximately 60% of patients in the present study underwent ^68^Ga-PSMA-PET/CT for staging upon initial diagnosis, and the results obtained from ^68^Ga-PSMA-PET/CT were used to determine the RT plan. Furthermore, the findings of the univariate analysis indicated that the use of ^68^Ga-PSMA-PET/CT had a significant impact on both FFBF and PCSS. This can be attributed to the improved identification of DM during the initial diagnosis, thereby enabling effective treatment interventions. In addition, the multivariable analysis demonstrated a significant correlation between ^68^Ga-PSMA-PET/CT staging and improved FFBF outcomes, highlighting the significance of accurate staging.

The survival benefit of dose escalation, either through the SIB technique or BT boost, has been demonstrated in recent randomized trials for patients with intermediate- and high-risk PCa [7, 8]. In addition, recent database trials have shown that BT boost to EBRT yields better results compared to EBRT alone or RP in patients with GS 9–10 PCa. [14, 16, 22, 25, 26]. Finally, Sandler et al. [19] reported 5‑year PCSS rates of 87%, 75%, and 94% for RP, EBRT, and EBRT with BT boost groups, respectively, in GS 10 PCa patients. The 5‑year DMFS rates for the RP, EBRT, and EBRT-BT groups were 64%, 62%, and 87%, respectively. Our findings align with those of previous research [19], highlighting the significant improvement in patient outcomes when applying dose escalation with the SIB technique. This emphasizes the importance of local control for reducing DM and PCa mortality in this specific patient population.

Recent randomized trials have shown the advantages of long-term ADT in comparison to short-term ADT or no ADT in high-risk PCa patients [6, 27, 28]. The MARCAP consortium has shown that the advantages of incorporating or extending ADT in the treatment of localized PCa with RT outweigh the benefits of escalating the RT dose [5, 11]. The results of this study also show the survival advantage of long-term ADT compared to ADT with a duration of less than 18 months in a patient population with a relatively high risk. Nevertheless, it is worth noting that patients with GS 10 tumors are at a high risk of developing DM. It is likely that future studies utilizing androgen receptor-targeted agents will provide evidence of the advantages of implementing aggressive hormonal therapy for this specific patient group. The STAMPEDE trial presented findings that support the notion that the use of combination therapy, specifically abiraterone and ADT, is associated with significantly higher rates of metastasis-free survival in patients with high-risk nonmetastatic PCa in comparison to the use of ADT alone [29]. The ENZARAD trial aims to assess the effectiveness of enzalutamide in comparison to non-steroidal anti-androgens with LHRHA in men who are scheduled to undergo RT for localized high-risk or node-positive PCa (ClinicalTrials.gov identifier: NCT 02446444). The ATLAS trial aims to evaluate the impact of combining apalutamide with LHRHA on the survival rates of high-risk PCa patients undergoing primary RT (ClinicalTrials.gov identifier: NCT02531516).

This study has limitations due to its retrospective design and potential selection bias. Our study is limited by a small patient population, and the duration of ADT varied according to clinical practice. In addition, our findings primarily pertain to needle biopsy specimens, which may not accurately reflect the actual Gleason score determined from RP specimens. Finally, our study was conducted at a single institution and would benefit from further validation to ensure a more precise assessment of clinical outcomes. Despite these limitations, this study also offers several advantages. For instance, it boasts a large sample size compared to previous series. Additionally, the study focused on a homogeneous patient population, all of whom underwent ADT and pelvic nodal irradiation with modern RT techniques.

## Conclusion

Our study revealed that GS 10 PCa exhibits a highly aggressive nature, as evidenced by the occurrence of DM in over 40% of patients throughout their clinical progression. While there is no agreement on how to treat this specific group of patients, our research indicates that a combination of long-term ADT and an increasing radiation dose at the primary tumor site leads to better clinical results, highlighting the significance of aggressive treatment in enhancing treatment outcomes. Furthermore, the use of ^68^Ga-PSMA-PET/CT for staging results in a substantial improvement in FFBF and PCSS when compared to staging using conventional imaging methods. This highlights the importance of improving the detection of DM in this very-high-risk patient group. As this study may be viewed as a hypothesis-generating investigation focusing on ^68^Ga-PSMA-PET/CT and radiation dose escalation, additional validation studies are needed to confirm its findings.
